# Comparative study of four sarcopenia-related risk prediction models for identifying SARC-F screening positivity in patients with colorectal cancer

**DOI:** 10.3389/fonc.2026.1867100

**Published:** 2026-08-11

**Authors:** Zhuoyun Li, Xiaoying Wang, Fang Liu, Yali Hu

**Affiliations:** 1 Gansu Provincial Hospital, Lanzhou, Gansu, China; 2 School of Nursing, Gansu University of Chinese Medicine, Lanzhou, Gansu, China

**Keywords:** colorectal cancer, nursing, predictive model, risk assessment, sarcopenia

## Abstract

**Objective:**

To compare the performance of four sarcopenia-related risk prediction models in identifying SARC-F screening positivity among patients with colorectal cancer, and to provide evidence for early inpatient screening.

**Methods:**

A total of 340 hospitalized patients with colorectal cancer were included. SARC-F screening positivity was defined as a Chinese version SARC-F score of ≥4. The Ren LSMI score, Lim LP-SMG score, Zhang Xuejing score, and Zhang Ying linear predictor were reconstructed according to their original formulas. Model performance was evaluated using receiver operating characteristic (ROC) curves, the DeLong test, 10-fold cross-validated recalibration, Brier score, calibration indices, and decision curve analysis.

**Results:**

Among the 340 patients, 52 were SARC-F screening positive, with a positivity rate of 15.29%. The AUC of Model 1 was significantly lower than those of the other three models, while no significant differences were observed among Models 2, 3, and 4. After recalibration, Model 3 had the lowest Brier score of 0.088, with a calibration intercept of −0.014 and a calibration slope of 0.909, and showed clinical net benefit across the threshold probability range of 1%–50%. Model 4 had the highest sensitivity, whereas Model 1 had relatively high specificity. Although Model 2 showed a high AUC, its recalibration slope was negative, suggesting that it was not suitable for direct prediction of this outcome.

**Conclusion:**

All four models showed some ability to identify SARC-F screening positivity. Among them, the Zhang Xuejing score demonstrated relatively stable overall performance and may serve as a preferred candidate tool for early inpatient screening. The Zhang Ying model may be useful for reducing missed cases, while the Ren LSMI score may help reduce false positives. Further validation in independent multicenter cohorts is warranted.

## 1 Introduction

Colorectal cancer (CRC) is a malignant tumor that develops in the colon or rectum. It is the third most common cancer, accounting for 9.6% of all cancer cases, and the second leading cause of cancer-related death, accounting for 9.3% of all cancer deaths (1–3). In China, the number of colorectal cancer cases surpassed that of gastric cancer in 2020, making CRC the second most common cancer. Moreover, nearly 80% of patients are diagnosed at intermediate or advanced stages at initial detection, and nearly half survive for less than 5 years (4, 5).

Sarcopenia was first proposed by Rosenberg in 1989. In 2010, the European Working Group on Sarcopenia in Older People (EWGSOP) formally defined sarcopenia as “a syndrome characterized by progressive and generalized loss of skeletal muscle mass and strength, with a risk of adverse outcomes such as physical disability, poor quality of life, and death” (6). Sarcopenia is an age-related, progressive, and generalized skeletal muscle disorder, mainly characterized by declines in skeletal muscle mass and function. It increases the risk of fractures, frailty, falls, disability, loss of independence, and death, and is common among patients with cancer (7–9). Owing to its high prevalence and serious adverse effects on health, sarcopenia has attracted increasing attention from researchers worldwide. Patients with colorectal cancer often experience digestive dysfunction, malabsorption, and malnutrition, which may further increase the risk of sarcopenia (10).

Risk prediction models are mathematical and statistical models that estimate the probability of a disease or outcome based on individual patient characteristics (11). Several risk models or scoring tools have been developed for sarcopenia-related conditions in patients with CRC. However, these models differ in their original outcomes, study populations, and predictor composition. Specifically, Ren et al (12) developed a prediction model for low skeletal muscle mass index (LSMI), Lim et al. (13) predicted low skeletal muscle gauge (SMG), Zhang Xuejing et al (14) constructed a risk score using an SARC-F-related outcome, and Zhang Ying (15) used sarcopenia defined according to the AWGS criteria as the outcome. These differences indicate that the risk estimates generated by different models cannot be directly interpreted as probabilities of the same disease outcome. To date, few studies have compared the relative performance of different published scores in the same hospitalized CRC population using a unified screening outcome. Therefore, in this single-center prospective observational cohort study, we reconstructed four published sarcopenia-related risk scores and used SARC-F ≥4 as the unified screening outcome. We compared their discriminatory ability and further evaluated their predictive accuracy, calibration performance, and clinical net benefit after being transported to the SARC-F screening-positive outcome through cross-validated recalibration.

## 2 Materials and methods

### 2.1 Study population

A total of 340 patients diagnosed with colorectal cancer and admitted to the Gansu Provincial Research Center for Anorectal Diseases between April 1 and August 1, 2025, were enrolled. The inclusion criteria were as follows (1): age ≥18 years (2); pathologically confirmed CRC (3); scheduled to undergo radical surgery for CRC (4); complete clinical data, including laboratory data; and (5) signed informed consent. The exclusion criteria were as follows (1): severe underlying diseases affecting nutritional status or muscle metabolism, such as severe hepatic or renal dysfunction or heart failure (2); severe infection or inflammatory conditions that could affect the assessment of sarcopenia (3); concomitant malignant tumors or multiple primary cancers (4); previous interventional nutritional therapy or exercise intervention (5); incomplete data or loss to follow-up; and (6) neuromuscular diseases or long-term bedridden status.

This was a prospective cohort study. According to a previous study (16), the prevalence of sarcopenia among patients with CRC was 33%. With an allowable error δ of 5%, the required sample size was estimated to be approximately 339 using the formula (17)n = p × (1 − p) × (1.96/δ)^2^. Finally, 340 patients were included in this study, of whom 52 were SARC-F screening positive. This study was approved by the Medical Ethics Committee of Gansu Provincial Hospital (approval number: 2025-458).

### 2.2 Methods

#### 2.2.1 Research tools

(1) A general patient information form (2); the LSMI model developed by Ren et al. (12)(hereafter referred to as Model 1), which includes age, body mass index (BMI), and grip strength as predictors (3); the model developed by Lim et al. (13)(hereafter referred to as Model 2), which includes sex, age, height, BMI, smoking history, tumor location, neutrophil-to-lymphocyte ratio (NLR), platelet-to-lymphocyte ratio (PLR), hemoglobin, and albumin-bilirubin (ALBI) score as predictors (4); the model developed by Zhang Xuejing et al. (14)(hereafter referred to as Model 3), which includes tumor stage, smoking history, drinking history, diabetes, nutritional status, and physical exercise as predictors; and (5) the model developed by Zhang Ying (15) (hereafter referred to as Model 4), which includes smoking history, diabetes, tumor stage, total protein, nutritional status, and physical activity as predictors.

#### 2.2.2 Screening tool for SARC-F positivity

In this study, the Chinese version of the Sarcopenia-Five questionnaire (SARC-F) (18) was used to screen for sarcopenia risk in patients with CRC. The SARC-F was translated and validated in Chinese by Huang Lijie et al. The scale consists of five items, each scored from 0 to 2, with a total score ranging from 0 to 10. A score of ≥4 indicates impaired physical function. This screening tool assesses patients using five easily obtainable items and determines the risk of sarcopenia through a scoring system. It is safe, convenient, inexpensive, and highly feasible for use by nursing staff (19). The scale has demonstrated good reliability and validity, with a Cronbach’s α coefficient of 0.803 and a test-retest reliability of 0.821, indicating that it can be used as an effective tool for early clinical screening of sarcopenia (18).

#### 2.2.3 Data collection methods

Data were collected using a combination of structured questionnaires and the electronic medical record system to ensure completeness and accuracy. The questionnaire included age, sex, body weight, height, tumor location, laboratory indicators, the SARC-F questionnaire, and other relevant clinical information. Age, sex, BMI, laboratory indicators, and other clinical variables were obtained within 24 hours after admission or from the first preoperative assessment. The SARC-F assessment was completed within 24 hours after admission.

Before data collection, all researchers received standardized training. Eligible participants were screened strictly according to the inclusion and exclusion criteria. After obtaining informed consent, the researchers conducted bedside assessments to evaluate the risk of sarcopenia-related functional impairment. Two researchers independently reviewed the patients’ electronic medical records, entered the relevant data, and cross-checked the entries. If a small amount of data was missing, multiple imputation was used to handle missing values, thereby ensuring data completeness and reliability.

To maintain consistency with the variable definitions used in the original models, nutritional risk in Model 3 was coded directly according to the original NRS-2002 score: NRS-2002 ≥3 was coded as 1, and NRS-2002 <3 was coded as 2. In the Zhang Ying model, the PG-SGA total score was classified as follows: ≤3 points as category A, indicating good nutritional status; 4–8 points as category B, indicating mild or suspected malnutrition; and ≥9 points as category C, indicating severe malnutrition. Category A was used as the reference group. According to the IPAQ-SF, a total MET-min/week of <600 was defined as low physical activity, 600 to <3000 as moderate physical activity, and ≥3000 as high physical activity.

#### 2.2.4 Reconstruction of risk scores

This study aimed to compare the performance of four published sarcopenia-related risk scores in identifying SARC-F screening positivity, rather than to develop new prediction models. Therefore, the original model coefficients were not re-estimated using the present cohort. Instead, each patient’s original continuous risk score was calculated according to the formulas, regression coefficients, intercepts, or nomogram-based total score rules reported in the original publications. The original outcomes, formula sources, variable coding, and variable mapping used in this study for each model are presented in **Table 1** and **Appendix 1**.

**Table 1:** Characteristics and reconstruction details of the four risk scores.

Item	Model 1	Model 2	Model 3	Model 4
Population	Patients with colorectal cancer (CRC)	Patients with colorectal cancer (CRC)	Patients with colorectal cancer (CRC)	Patients with colorectal cancer (CRC)
Model Type	Logistic regression + nomogram	LASSO-based LP-SMG model	Logistic regression + nomogram	Logistic regression + nomogram
Objective	Development and validation of a sarcopenia risk prediction model in CRC patients	Development and validation of a sarcopenia risk prediction model in CRC patients	Development and validation of a sarcopenia risk prediction model in CRC patients	Development and validation of a sarcopenia risk prediction model in CRC patients
Sample Size	1692	1094	450	359
Calibration Method	Hosmer–Lemeshow test + calibration curve	Primarily ROC and DCA analyses	Hosmer–Lemeshow test + calibration curve	Hosmer–Lemeshow test + calibration curve
Source of original formula	Nomogram total score formula	LASSO-logistic regression coefficients	Original continuous risk score	Binary logistic regression table
Main variables	Age; BMI; grip strength	Sex, age, height, BMI, smoking, tumor location, NLR, PLR, hemoglobin, ALBI	Tumor stage, smoking, drinking, diabetes, NRS-2002, physical exercise	Tumor stage, smoking, diabetes, total protein, PG-SGA, physical activity
Reconstructed according to the original formula	Yes	Yes	Yes	Yes

Model 1 was calculated according to the LSMI total score formula reported by Ren et al., by directly entering age, BMI, and grip strength. Model 2 was calculated according to the LP-SMG formula published by Lim et al., with variable coding and unit conversion performed as required in the original study. Model 3 was calculated using the formula reported in the main text of the study by Zhang Xuejing et al., with all variables coded according to the original definitions. Model 4 was calculated according to the logistic regression formula reported by Zhang Ying.

Because the four source models differed in study populations, predictor composition, and original prediction outcomes, this study only compared their performance after being transported to the SARC-F screening-positive outcome. During the analysis, the timing of variable measurement, unit conversion, and coding rules were standardized; however, the original model coefficients were not modified, and the different original outcomes were not regarded as equivalent to the same diagnostic outcome of sarcopenia.

#### 2.2.5 Statistical analysis

Data were analyzed using SPSS 25.0 and R 4.5.1. Categorical variables were described as frequencies and percentages. Normally distributed continuous variables were expressed as mean ± standard deviation, whereas non-normally distributed continuous variables were expressed as median and interquartile range. ROC curves for each prediction model were plotted using the pROC package in R. The area under the curve (AUC), sensitivity, and specificity were calculated, and paired DeLong tests were used to compare the AUCs of the four scores. The Hosmer–Lemeshow goodness-of-fit test was used to preliminarily assess the agreement between predicted and observed values, with P > 0.05 indicating no significant lack of fit.

Because the four original models differed in their prediction outcomes and not all of them directly provided predicted probabilities for SARC-F screening positivity, 10-fold cross-validated logistic recalibration was further performed to generate out-of-fold predicted probabilities. These probabilities were then used to calculate the Brier score, calibration intercept, calibration slope, and decision curve analysis. A lower Brier score indicates a smaller prediction error, and the ideal values of the calibration intercept and calibration slope are 0 and 1, respectively. A total of 2,000 paired bootstrap resamples were used to calculate the 95% confidence intervals for the Brier score and calibration indices, and to compare the Brier score difference between each score and the best-performing score. The threshold probability range for decision curve analysis was set at 1%–50%. All statistical tests were two-sided, and P < 0.05 was considered statistically significant.

## 3 Results

### 3.1 Baseline characteristics of patients

A total of 340 eligible patients with CRC were included in this study. Among them, 52 were SARC-F screening positive, with a screening positivity rate of 15.29%. Among the SARC-F screening-positive patients, 21 were male and 31 were female. The baseline characteristics of the patients are shown in **Table 2**.

**Table 2:** Baseline characteristics of patients.

Variable	SARC-F-positive group (n = 52)	SARC-F-negative group (n = 288)	Test statistic	P
Age [years, Median (P25, P75)]	69.0 (57.5, 72.5)	60.0 (53.0, 66.0)	Z = 4.264	< 0.001
BMI [kg/m^2^, Median (P25, P75)]	21.6 (18.9, 23.9)	22.5 (20.5, 25.0)	Z = -2.079	0.038
Sex [n (%)]			χ^2^ = 10.164	0.001
Male	21 (40.38%)	184 (63.89%)		
Female	31 (59.62%)	104 (36.11%)		
History of alcohol consumption [n (%)]			–	0.041
No	10 (19.23%)	90 (31.25%)		
Yes	42(80.77%)	198 (68.75%)		
History of smoking [n (%)]			–	0.011
Yes	42 (80.77%)	170 (59.03%)		
No	10 (19.23%)	118 (40.97%)		
Diabetes mellitus [n (%)]			χ^2^ = 2.286	0.131
No	47 (90.38%)	275 (95.49%)		
Yes	5 (9.62%)	13 (4.51%)		
Tumor location [n (%)]			χ^2^ = 5.874	0.053
Rectum	39 (75.00%)	194 (67.36%)		
Colon	9 (17.31%)	86 (29.86%)		
Rectosigmoid junction	4 (7.69%)	8 (2.78%)		

The *Z* values represent standardized test statistics from the Mann–Whitney U test (used for non-normally distributed continuous variables); all other values represent Chi-square (χ^2^) test statistics for categorical variables.

### 3.2 Discriminatory ability of the four risk scores for SARC-f screening positivity

**Figure 1:**
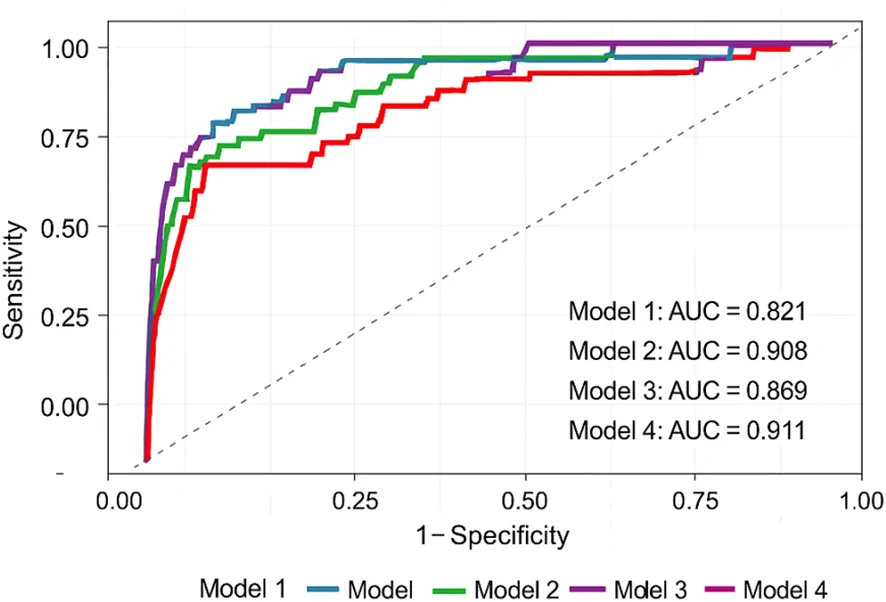
ROC curves of the four risk scores for identifying SARC-F screening positivity in patients with CRC.

The AUC values were 0.821 for Model 1, 0.908 for Model 2, 0.869 for Model 3, and 0.911 for Model 4. Pairwise comparisons using the paired DeLong test showed that the *AUC* of Model 1 was significantly lower than those of Models 2, 3, and 4, whereas no statistically significant differences were observed among Models 2, 3, and 4. These results suggest that, except for Model 1, the other three models had comparable discriminatory ability for identifying SARC-F screening positivity. The risk identification performance of the four models for SARC-F screening positivity in patients with CRC is shown in **Table 3**. ROC curves were plotted to compare the ability of each model to identify SARC-F screening positivity, as shown in **Figure 1**.

**Table 3:** Predictive performance of the four risk scores for SARC-F screening positivity in patients with CRC.

Model	AUC	95%CI	Optimal Cut-off value	Sensitivity	Specificity	Youden
Model 1	0.821	(0.751, 0.890)	5.087	0.654	0.910	0.564
Model 2	0.908	(0.858, 0.958)	4.963	0.808	0.882	0.690
Model 3	0.869	(0.809, 0.928)	4.456	0.712	0.910	0.621
Model 4	0.911	(0.870, 0.952)	2.860	0.923	0.753	0.677

**Figure 2:**
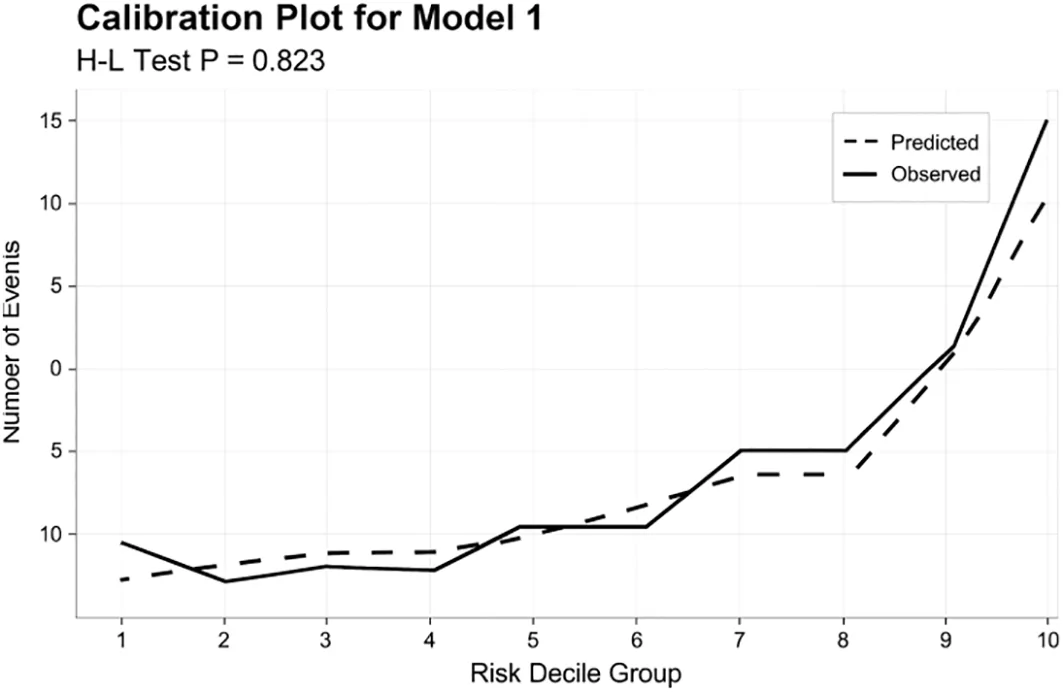
Hosmer–Lemeshow observed-versus-expected plot for Mode l.

**Figure 3:**
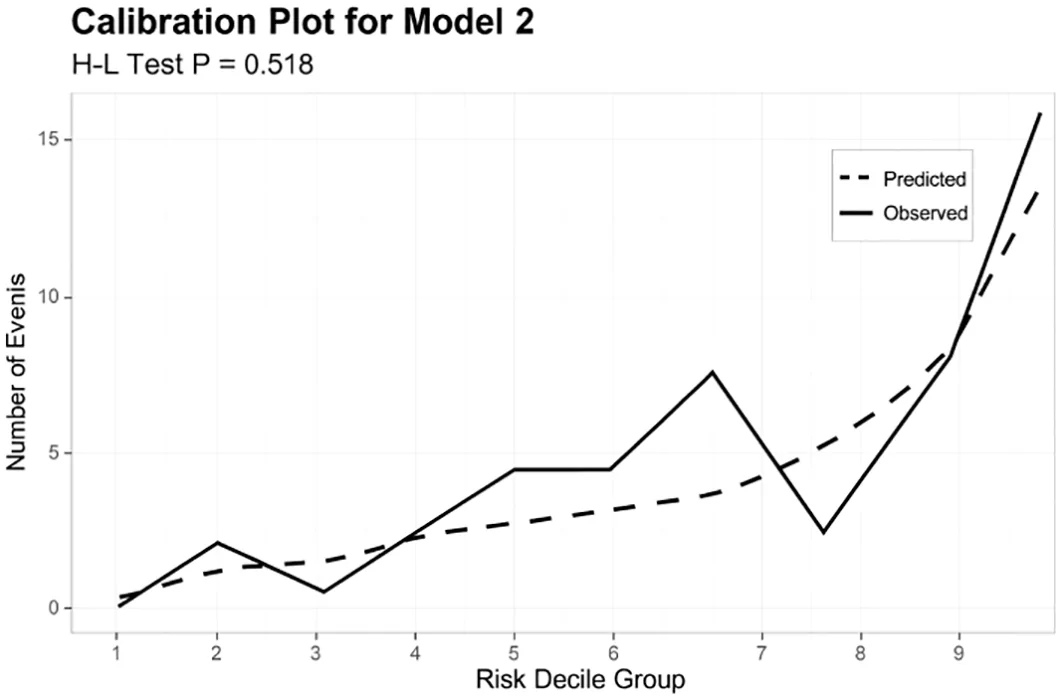
Hosmer–Lemeshow observed-versus-expected plot for Model 2.

**Figure 4:**
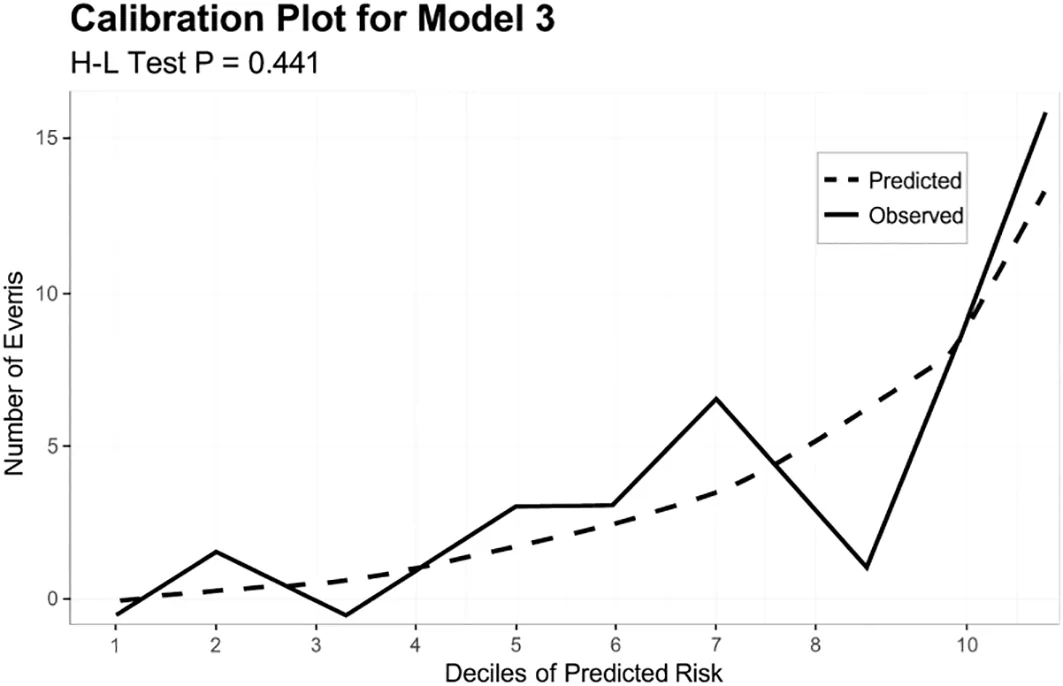
Hosmer–Lemeshow observed-versus-expected plot for Model 3.

**Figure 5:**
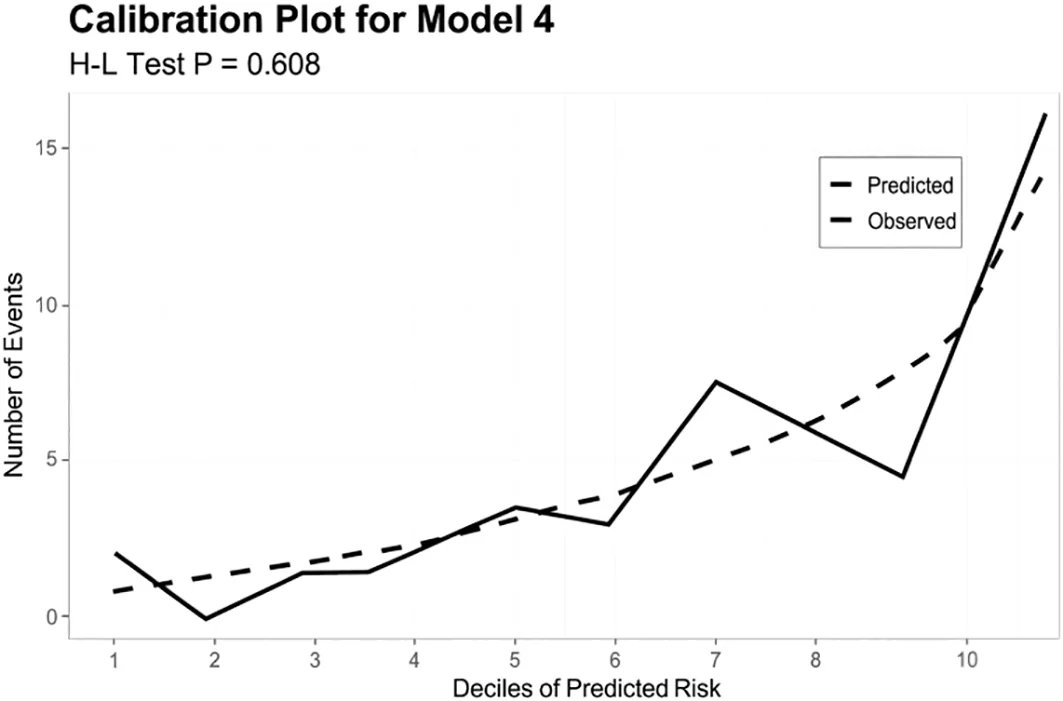
Hosmer–Lemeshow observed-versus-expected plot for Model 4.

The Hosmer–Lemeshow goodness-of-fit test was used to evaluate the agreement between the predicted and observed values of the four models. The results were as follows: Model 1, *χ*^2^ = 3.05, *P* = 0.823; Model 2, *χ*^2^ = 6.46, *P* = 0.518; Model 3, *χ*^2^ = 7.28, *P* = 0.441; and Model 4, *χ*^2^ = 6.23, *P* = 0.608, as shown in **Figures 2**–**5**. The Hosmer–Lemeshow test showed no significant lack of fit, which only indicates that no obvious lack of fit was observed in this sample under the current grouping strategy. Model calibration still needs to be comprehensively assessed using calibration plots, Brier scores, calibration intercepts, and calibration slopes.

### 3.3 Predictive accuracy and calibration performance after recalibration

**Figure 6:**
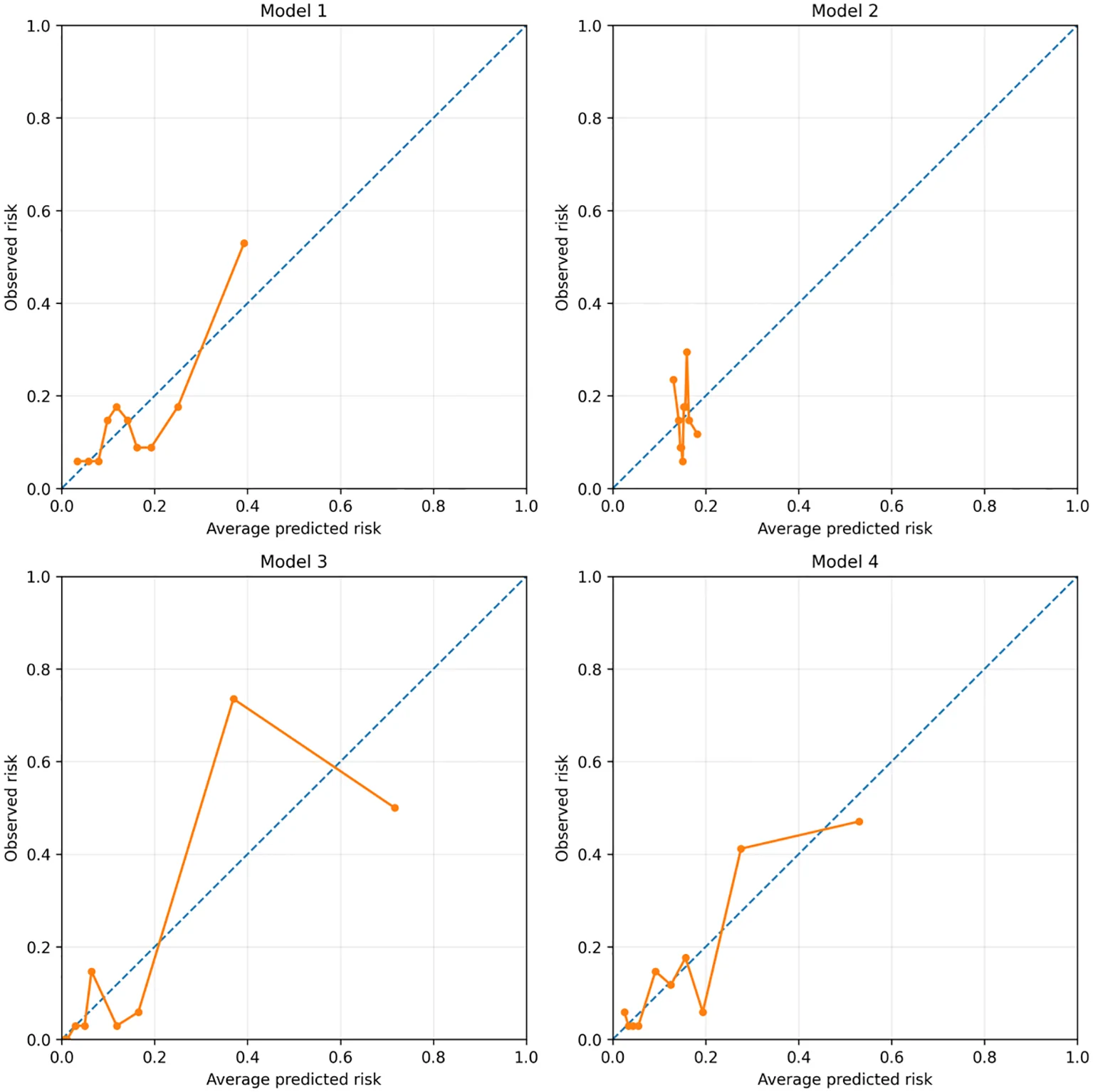
Calibration curves after cross-validated recalibration.

Based on the out-of-fold predicted probabilities from 10-fold cross-validation, Model 3 had the lowest Brier score, which was significantly lower than those of Model 1, Model 2, and Model 4. The calibration intercept and calibration slope of Model 3 were −0.014 and 0.909, respectively. The calibration indices of the linear predictor of Model 4 and the score of Model 1 were also close to the ideal values. In contrast, Model 2 had a negative recalibration slope, suggesting that its original score was inversely associated with SARC-F screening positivity and was therefore not suitable as an effective tool for identifying this outcome. The results are presented in **Table 4**. Calibration curves of the four models after cross-validated recalibration are shown in **Figure 6**.

**Table 4:** Cross-validated recalibration performance of the four risk scores.

Model	Recalibration direction	Brier score (95% CI)	ΔBrier vs. best score (P value)	Calibration intercept (95% CI)	Calibration slope (95% CI)	DCA benefit range
Model 1	Positive	0.117 (0.094, 0.142)	0.029 (0.007, 0.051;P=0.006)	0.003 (−0.337, 0.289)	0.973 (0.551, 1.475)	1%–3%; 7%–48%
Model 2	Negative	0.130 (0.104, 0.155)	0.042 (0.020, 0.064;P<0.001)	0.000 (−0.325, 0.257)	−1.497 (−4.557, 1.640)	Only at isolated threshold points
Model 3	Positive	0.088 (0.068, 0.109)	—	−0.014 (−0.445, 0.343)	0.909 (0.673, 1.309)	1%–50%
Model 4	Positive	0.107 (0.084, 0.130)	0.019 (0.004, 0.034;P=0.009)	−0.001 (−0.357, 0.292)	0.957 (0.684, 1.313)	2%; 5%–50%

A lower Brier score indicates better performance. The ideal values of the calibration intercept and calibration slope are 0 and 1, respectively. The DCA benefit range refers to the main threshold probability intervals in which the net benefit was higher than both the “treat-all” and “treat-none” strategies.

### 3.4 Decision curve analysis

**Figure 7:**
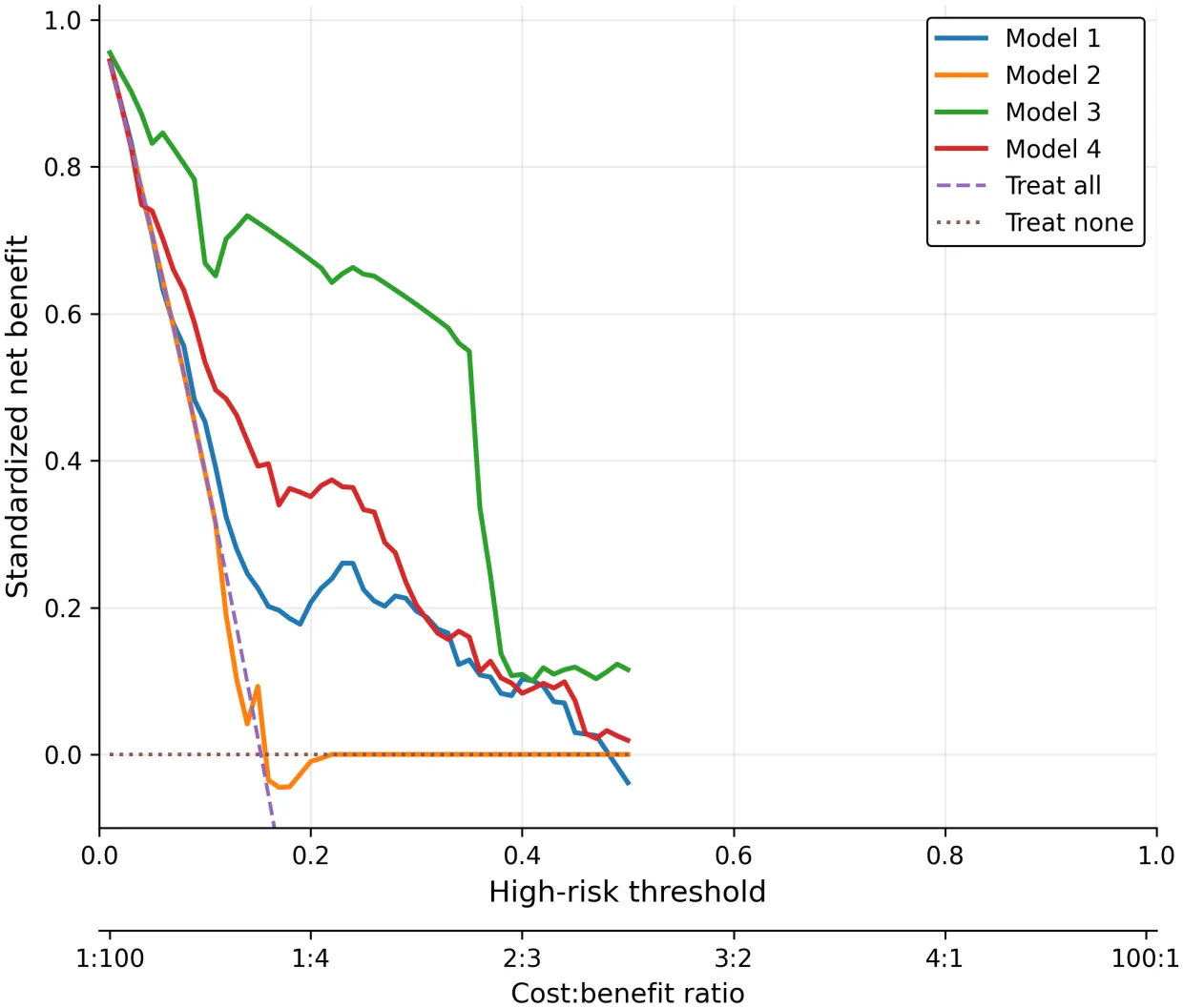
Decision curve analysis after cross-validated recalibration.

Within the threshold probability range of 1%–50%, Model 3 showed a consistently higher net benefit than both the strategy of “all patients undergoing further sarcopenia assessment” and the strategy of “no patients undergoing additional assessment,” suggesting that this score may help identify patients who require further objective assessment for sarcopenia. Model 4 showed positive net benefit across most threshold probabilities from 5% to 50%. Model 1 demonstrated some net benefit within the range of 7%–48%, whereas Model 2 showed net benefit only at isolated threshold points and did not demonstrate stable clinical utility, as shown in **Figure 7**.

## 4 Discussion

### 4.1 SARC-F screening positivity in patients with CRC

In recent years, the number of patients with colorectal cancer complicated by sarcopenia has gradually increased. Previous studies have reported that the prevalence of sarcopenia among patients with colorectal cancer is approximately 19.4% (20). In the present study, the Chinese version of the SARC-F questionnaire was used to assess 340 hospitalized patients with CRC, among whom 52 were SARC-F screening positive, corresponding to a positivity rate of 15.29%. Previous studies (21, 22) have shown considerable variation in the prevalence of sarcopenia among patients with CRC. The rate observed in this study was similar to that reported by Souza et al (23), but differed from that reported by J. He et al (16). These discrepancies may be related to differences in study populations, disease stage, and nutritional status. In addition to differences in patient characteristics, the use of SARC-F as the screening tool in the present study may also have contributed to the variation. It should be noted that this study used SARC-F ≥4 as the unified screening outcome, which reflects sarcopenia-related functional impairment and screening-positive risk. Therefore, the findings are more applicable to early inpatient screening for sarcopenia risk.

Age has been widely recognized as a key factor associated with sarcopenia (24). In the present study, patients with SARC-F screening positivity were older, had lower BMI, and had higher proportions of women, smoking history, and drinking history. Older age and lower BMI generally indicate reduced physiological reserve and impaired muscle function, which may make patients more likely to experience SARC-F-related manifestations such as limited mobility, reduced walking ability, and difficulty rising from a chair. This may be associated with the progressive atrophy of type II muscle fibers and motor neurons that occurs with aging. During the early stage of hospitalization for patients with CRC, healthcare professionals should pay particular attention to older patients, those with lower BMI, and those with unhealthy lifestyle factors, and should conduct timely assessments of nutritional status, physical activity, and muscle function.

### 4.2 Comparison of the predictive value of the four risk scores for SARC-F screening positivity

This study systematically compared the risk identification performance of four different models for SARC-F screening positivity in patients with CRC. In terms of discrimination, Model 4 had an AUC of 0.911, followed by Model 2 with an AUC of 0.908, Model 3 with an AUC of 0.869, and Model 1 with an AUC of 0.821. The AUROC values of all four models were greater than 0.8, indicating that they all had relatively strong ability to identify SARC-F screening positivity in patients with CRC. Model 1 showed the lowest discriminatory ability among the four models, but it had relatively high specificity. This may be because the model was applied to a new dataset, and differences in sample characteristics from those in the internal validation cohort may have led to reduced sensitivity. In general, lower sensitivity is often accompanied by higher specificity, which may explain the increased specificity of the model in the new dataset (25).

In terms of goodness of fit, the Hosmer–Lemeshow (H-L) test showed no significant lack of fit for all four models (P > 0.05). The differences between the observed and predicted values were relatively small, suggesting that no obvious lack of fit was observed in this sample. However, the H-L test is affected by grouping strategy and sample size and cannot independently prove good model calibration. Therefore, this study further performed recalibration using out-of-fold predicted probabilities from 10-fold cross-validation and comprehensively evaluated model performance by combining the Brier score, calibration intercept, calibration slope, and decision curve analysis.

Model 3 showed the most stable performance among the four models. It had the lowest Brier score of 0.088, which was significantly lower than those of Models 1, 2, and 4, indicating the smallest overall error between predicted probabilities and actual SARC-F screening outcomes. The calibration intercept and calibration slope of Model 3 were −0.014 and 0.909, respectively, both close to the ideal values. In addition, Model 3 showed favorable clinical net benefit across the threshold probability range of 1%–50%. The variables included in this model are closely related to nutritional status, physical activity, and functional impairment in patients with CRC, and are therefore consistent with the functional sarcopenia-related risk reflected by SARC-F. Thus, Model 3 may be preferentially considered for early inpatient screening in patients with CRC. In clinical practice, patients identified as high risk by Model 3 may undergo further assessment of grip strength, physical activity, nutritional risk, and CT-derived skeletal muscle mass.

Model 4 had the highest AUC, and its sensitivity at the optimal cut-off value was 0.923, suggesting strong ability to identify patients with SARC-F screening positivity and a relatively low risk of missed cases. After recalibration, Model 4 had a Brier score of 0.107, a calibration intercept of −0.001, and a calibration slope of 0.957, all of which were close to the ideal values. It also showed clinical net benefit across the main threshold probability range of 5%–50%. The indicators included in Model 4 cover disease status, nutritional status, and physical activity, and are relatively easy to obtain in clinical practice. Therefore, this model may be suitable for scenarios in which minimizing missed cases is a priority. For older patients, patients with low BMI, poor nutritional status, or reduced physical activity, preliminary screening using Model 4 may help identify those with possible sarcopenia-related functional impairment at an early stage.

Model 1 had an AUC of 0.821, which was relatively lower than those of the other three models. However, its calibration intercept was 0.003 and its calibration slope was 0.973, suggesting good agreement between the recalibrated predicted probabilities and the observed outcomes. Model 1 also had relatively high specificity, making it more suitable for situations in which reducing false positives and avoiding excessive assessment are priorities. Patients predicted to be at high risk may be prioritized for further muscle function or nutritional assessment, whereas patients predicted to be at low risk may receive routine follow-up according to clinical circumstances.

Model 2 had an AUC of 0.908, indicating apparently high discriminatory ability. However, its recalibration slope was −1.497, suggesting an inverse relationship between the original linear predictor of Model 2 and SARC-F screening positivity in the present study. Model 2 had the highest Brier score of 0.130, and decision curve analysis showed clinical net benefit only at isolated threshold points. Therefore, although Model 2 was able to distinguish between screening-positive and screening-negative patients in the ROC analysis, its original risk direction and probability interpretation are not directly applicable for predicting SARC-F screening positivity. These findings suggest that before clinical application, the score direction should be further adjusted, the cut-off value should be redefined, and its stability should be validated in an independent sample.

## 5 Conclusions

This study compared the performance of four published sarcopenia-related risk scores in identifying SARC-F screening positivity in a single-center cohort of hospitalized patients with CRC. The results showed that, after recalibration, Model 3 had a lower Brier score and relatively stable net benefit in decision curve analysis, suggesting that it may serve as a preferred candidate tool for early inpatient identification of SARC-F screening positivity. Model 4 had higher sensitivity and may be useful in initial screening scenarios where reducing missed cases is prioritized. Model 1 had higher specificity and may serve as an auxiliary tool for reducing false positives. Early identification of high-risk patients may help provide preliminary evidence for subsequent nutritional assessment, exercise intervention, rehabilitation guidance, and perioperative management.

This study has several limitations. First, SARC-F ≥4 was used as the unified outcome. Although SARC-F is convenient for rapidly identifying the risk of sarcopenia-related functional impairment in the early stage of hospitalization, it is a self-reported screening tool and does not directly reflect skeletal muscle mass. Therefore, this study evaluated the ability of the four scores to identify the risk of SARC-F screening positivity in patients with CRC. Second, this was a single-center study with a limited sample size, and only 52 patients were SARC-F screening positive. The stability and generalizability of the findings therefore require further validation in multicenter independent cohorts using objective indicators such as CT-derived muscle mass, grip strength, and physical performance. Future multicenter studies should incorporate CT-derived skeletal muscle mass, grip strength, and physical performance measures to validate the predictive value of these scores for sarcopenia diagnosis and related adverse outcomes.

## Data Availability

The raw data supporting the conclusions of this article will be made available by the authors, without undue reservation.
