# Supplementary material for: Comparative study of four sarcopenia-related risk prediction models for identifying SARC-F screening positivity in patients with colorectal cancer
Source: Front Oncol. 2026 Aug 11;16:1867100. doi: 10.3389/fonc.2026.1867100 (PMC13503187; doi:10.3389/fonc.2026.1867100)
Supplement: Supplementary file 2 [file Table1.docx]

Supplementary Material

# 1 Supplementary Data

Reconstruction Formulas and Variable Coding for the Four Risk Scores

To improve reproducibility, the formulas used to calculate the four risk scores in this study are listed below. All continuous variables were entered using the original units specified in each model. LP denotes the linear predictor.

## Model 1: Ren LSMI Score

S_Ren = 0.564930 × age (years) − 4.545455 × BMI (kg/m²) − 0.593798 × grip strength (kg) + 187.9656

## Model 2: Lim LP-SMG

LP_Lim = −12.532668911 + 2.647527908 × male + 0.088957259 × age (years) + 3.113010884 × height (m) − 0.028209671 × BMI (kg/m²) + 0.218438466 × smoking history − 0.188171134 × rectal cancer − 0.042050519 × NLR + 0.002422926 × PLR − 0.010913238 × hemoglobin (g/dL) + 0.178875872 × ALBI

**Variable coding:** male = 1 and female = 0; smoking history: yes = 1 and no = 0; rectal cancer = 1, and colon/ileocecal cancer = 0. Hemoglobin was entered in g/dL.

ALBI = 0.66 × log10[total bilirubin (μmol/L)] − 0.085 × albumin (g/L)

The original predicted probability was calculated as:

P_Lim = 1 / [1 + exp(−LP_Lim)]

## Model 3: Zhang Xuejing Score

Z_XJ = 6.681 + 0.035 × tumor stage + 0.573 × smoking history + 0.317 × drinking history + 0.889 × diabetes − 0.817 × nutritional status − 0.563 × physical exercise

**Variable coding:** tumor stage I–II = 1, stage III = 2, and stage IV = 3; smoking history, drinking history, and diabetes: yes = 1 and no = 0; NRS-2002 ≥3 = 1 and NRS-2002 <3 = 2; low, moderate, and high physical activity were coded as 1, 2, and 3, respectively.

## Model 4: Zhang Ying Linear Predictor

LP_ZY = 1.072 + 1.655 × tumor stage + 2.671 × smoking history + 1.701 × diabetes − 0.074 × total protein (g/L) + 0.347 × PG-SGA_B + 2.438 × PG-SGA_C − 1.845 × moderate physical activity − 2.979 × high physical activity

**Variable coding:** tumor stage III/IV = 1 and stage I/II = 0; smoking history: yes = 1 and no = 0; diabetes: yes = 1 and no = 0. For PG-SGA classification, a score ≤3 was classified as category A and used as the reference group, 4–8 as category B, and ≥9 as category C. PG-SGA_B and PG-SGA_C were entered as dummy variables. Low physical activity was used as the reference group, while moderate physical activity and high physical activity were entered as dummy variables.

The original predicted probability was calculated as:

P_ZY = 1 / [1 + exp(−LP_ZY)]
